# Alendronate modified mPEG-PLGA nano-micelle drug delivery system loaded with astragaloside has anti-osteoporotic effect in rats

**DOI:** 10.1080/10717544.2022.2086942

**Published:** 2022-07-22

**Authors:** Yanhai Xi, Weiheng Wang, Liang Ma, Ning Xu, Changgui Shi, Guohua Xu, Hailong He, Wenming Pan

**Affiliations:** a Department of Orthopedics, Spine Surgery, The Second Affiliated Hospital of Naval Medical University, Shanghai, China; b Minimally invasive Spinal Surgery department, The Sixth Affiliated Hospital of Xinjiang Medical University, Urumqi, China; c Department of Spine Surgery, The Affiliated Changshu Hospital of Xuzhou Medical School, The Second People's Hospital of Changshu, Changshu, China

**Keywords:** Astragaloside, mPEG-PLGA, targeted therapy

## Abstract

Astragaloside (AS) has an anti-osteoporotic effect, but its poor water solubility and low bioavailability limit its application. In this study, a novel nano-carrier with bone targeting was prepared by modifying mPEG-PLGA with alendronate (AL) before incorporation into astragaloside nano-micelles (AS-AL-mPEG-PLGA) to enhance the oral bioavailability, bone targeting and anti-osteoporosis effect of AS. The release behavior of AS-AL-mPEG-PLGA in vitro was investigated via dialysis. The pharmacokinetics of AS-AL-mPEG-PLGA was studied in Sprague-Dawley (SD) rats. The cytotoxicity of AS-AL-mPEG-PLGA in vitro (via MTT method), coupled with bone targeting ability in vitro and in vivo were evaluated. The therapeutic effects of free AS and AS-AL-mPEG-PLGA (ELISA, micro-CT, H&E staining) were compared in osteoporotic rats. AS-AL-mPEG-PLGA with smaller particle size (45.3 ± 3.8 nm) and high absolute zeta potential (−23.02 ± 0.51 mV) were successfully prepared, wherein it demonstrated higher entrapment efficiency (96.16 ± 0.18%), a significant sustained-release effect for 96 h and acceptable safety within 10–200 μg/mL. AS-AL-mPEG-PLGA could enhance the hydroxyapatite affinity and bone tissue concentration of AS. The relative bioavailability of AS-AL-mPEG-PLGA was 233.90% compared with free AS. In addition, the effect of AS in reducing serum levels of bone metabolism-related indicators, restoring the bone microarchitecture and improving bone injury could be enhanced by AS-AL-mPEG-PLGA. AS-AL-mPEG-PLGA with small particle size, good stability, remarkable sustained-release effect, safety and bone targeting was successfully constructed in this experiment to potentially improve the oral bioavailability and anti-osteoporosis effect of AS. Thus, AS-AL-mPEG-PLGA may be a promising strategy to prevent and treat osteoporosis.

## 1. Introduction

Osteoporosis, as a systemic bone metabolic disease, leads to severe complications such as brittle fracture, which seriously affects the quality of life of middle-aged and elderly people, and has become one of the global health problems (Maraka & Kennel, 2015). Usually, females are affected more by osteoporosis than males (Wang et al., 2019) probably because of their lighter bones. It is characterized by the destruction of bone homeostasis and loss of bone tissue, leading to fracture and bone pain (Neer et al., 2001). Destruction of bone microstructure in osteoporotic patients was associated with expression of bone gla protein (BGP), tartrate-resistant acid phosphatase-5b (TRACP-5b), C-terminal telopeptides of type I collagen (CTX-1) and inflammatory factor interleukin-6 (IL-6) (Godfrin-Valnet et al., 2014; Zhang et al., 2015; ). At present, the main drugs used in the treatment of osteoporosis are bone resorption inhibitors (such as estrogen, calcitonin, and diphosphonate) and bone formation promoters (namely fluoride, parathyroid hormone, strontium salt) (Wei et al., 2014). However, the aforementioned osteoporotic treatment methods always induce serious side effects such as heart attack and jaw necrosis (Cai et al., 2017). Therefore, developing a more effective and safer therapy drug for the treatment of osteoporosis is urgent.

Nowadays, a variety of natural active ingredients isolated from traditional Chinese medicine, such as osthol (Ming et al., 2011), icariin (Sun et al., 2019), ginsenoside Rg1 (Gong et al., 2006) and Panax notoginseng saponins (Li et al., 2011) have been proposed for the treatment of osteoporosis. *Astragalus membranaceus* is an important traditional Chinese medicine for the prevention and treatment of osteoporosis (Ou et al., 2019). Astragaloside (AS) is the main component of *A. membranaceus* (Bian et al., 2011). Guo et al. (2019) discovered that AS could promote the proliferation and migration of osteoblasts via the hedgehog signaling pathway. Another study (Sun et al., 2020b) has found that AS promoted differentiation of bone marrow mesenchymal stem cells, which may be a potential target for the treatment of osteoporosis. Unfortunately, low solubility in biological fluids and poor bioavailability after oral administration are the major limitations to its clinical application (Qing et al., 2019). Zhang indicated that the absolute bioavailability of AGS after p.o. the administration was found to be only 7.4% (Zhang et al., 2007). Previously, some preparations have been developed to overcome these obstacles, namely cyclodextrin inclusion compounds (Chen & Gu, 2009) and solid lipid nanoparticle-enriched hydrogel (Chen et al., 2013) [19]. However, the drug circulation extension *in vivo* and treatment of osteoporosis improvement has not been realized. Therefore, a novel AS-loaded nano drug delivery system is particularly needed for the oral bioavailability increase and treatment of osteoporosis. At present, studies (Jiang et al., 2015; Nie et al., 2017) have shown that polymeric micelles are an ideal nano-carrier with the capability to improve the solubility and bioavailability of osteoporotic drugs.

Micelles, as an important strategy to promote the dissolution of hydrophobic drugs, have been successfully used to improve the oral bioavailability of various compounds (Sun et al., 2020a; Wang et al., 2019). In particular, polymeric micelles are core-shell structures formed by the self-assembly of amphiphilic block polymers in the water environment. The hydrophobic end forms a core, while the hydrophilic end forms a shell, amid the former possibly containing hydrophobic drugs (Xie et al., 2019). Amphiphilic block polymers have been widely used in drug delivery systems due to their self-assembling properties. The more commonly used amphiphilic block polymers are PEG-PCL, PEG-PLGA and PEG-PLA (Daman et al., 2015; Grossen et al., 2017; Xie et al., 2018). MPEG-PLGA is an amphiphilic block polymer with a hydrophilic end of PEG and a hydrophobic end of PLGA (Zhang et al., 2014). PEG is a relatively frequently-used hydrophilic polymer, while PLGA is also a commonly used hydrophobic block, which has been applied in many drug delivery systems. Both PEG and PLGA are FDA-approved vehicles for medicinal use (Bakhaidar et al., 2020). PEG can avoid the endocytosis of reticuloendothelial cells and play a long circulation role. It can reduce the scavenging effect of the kidney, prolong the half-life and water solubility of the drug (Pasut & Veronese, 2009). Also, PEG has good biocompatibility coupled with enhanced permeability retention (EPR) effect and can achieve the purpose of passive targeting (Cosco et al., 2015). Compared with other polymers, PLGA can be degraded into nontoxic substances *in vivo* and has excellent biocompatibility (Manabe et al., 2015). Based on the above advantages, mPEG-PLGA was selected as the polymer skeleton in this study to prepare the polymeric micelles. Modifying PEG-PLGA with small molecular compounds with bone targeting function can better increase the bone targeting of this amphiphilic material. In 1986, Pierce & Waite (1987) found that some compound molecules have the trend of deposition in bone and incorporation of HAp, that is, they have the effect of “bone targeting.” Pierce’s discovery has led to extensive research on bone targeting molecules. At present, the reported bone targeting molecules include bisphosphonates, tetracyclines, amino acid oligopeptides, Rhubarb Anthraquinones, polypropionate esters, other small molecules, etc (Cai et al., 2012; Chang et al., 2016; Jiang et al., 2014; Neale et al., 2009; Wang et al., 2015). Bisphosphonates have significant bone targeting, wherein they are stable to enzymes and many chemicals (Wang et al., 2005), coupled with other compounds without changing their structure, amid being the most widely used bone targeting ligands at present. The oxygen atoms on the two phosphonic acid groups in the Al structure can chelate with calcium ions in HAP, thus showing a strong affinity for bone and calcified tissues (Katsumi et al., 2015), wherein the bone uptake rate can be as high as 50%–60%. Ye et al. (2015) modified doxorubicin (DOX) with AL and PEG to prepare ALN-PEG-hyd-DOX self-assembled pH sensitive micelles for the treatment of bone metastases in mice. The experimental results show that the prepared micelles exhibited good bone targeting, improved the antitumor activity of DOX and reduced the damage of tumor to bone tissue.

Alendronate (AL) sodium modified mPEG-PLGA was firstly utilized as a material in this study to encapsulate AS via micellar formation (AS-AL-mPEG-PLGA) to purposively achieve sustained release, improved oral bioavailability *in vivo*, and enhanced osteoporotic symptoms in rats. The prepared AS-AL-mPEG-PLGA were characterized by particle size, zeta potential, entrapment efficiency and morphology. The release behavior, cytotoxicity and bone targeting ability of AS-AL-mPEG-PLGA *in vitro* were also studied. In addition, the oral bioavailability and anti-osteoporotic activity of AS-AL-mPEG-PLGA were investigated *in vivo.*

## 2. Materials and methods

### 2.1. Materials

MPEG2000-PLGA18000 (50/50) was provided by A.V.T. Pharmaceutical Tech Co., Ltd (Shanghai, China). N, N-carbonyl diimidazole (CDI) and N. N-dimethylformamide (DMF) were bought from Macklin Biochemical Industry Co., Ltd (Shanghai, China). Aladdin Indust., Corp. (Shanghai, China) supplied tetrabutylammonium hydroxide and AS. AL sodium trihydrate was purchased from Shanghai Zhenzhun Biotechnology Co., Ltd (Shanghai, China). Invitrogen (Carlsbad, CA, USA) supplied 3-(4,5-dimethylthiazol-2-yl)-2,5-diphenyl-tetrazolium bromide (MTT), RPMI-1640 medium, fetal bovine serum (FBS). HPLC methanol and absolute ethanol were of analytical grade and were obtained from Sinopharm Chemical Reagent Co., Ltd (Shanghai, China). TRACP 5 b, BGP, IL-6 and CTX-1 ELISA kits were bought from Nanjing Jiancheng Bioeng. Inst., (Nanjing, China).

### 2.2. Cells and animals

Mouse osteoblast MC3T3-E1 cells were purchased from the cell bank of the Academy of Science (Shanghai, China). The cells were cultured in RPMI-1640 complete medium at 37 °C and 5% CO2 humidification. The Sprague-Dawley (SD) rats (200 ± 20 g) were provided by the laboratory and animal research center of Changzheng Hospital, Second Military Medical University, (Shanghai, 200003, China). The review and approval of protocol for all animal experiments complied with the committee for the use and care of animals.

### 2.3. Synthesis of alendronate modified mPEG-PLGA (AL-mPEG-PLGA)

**Figure 1.**
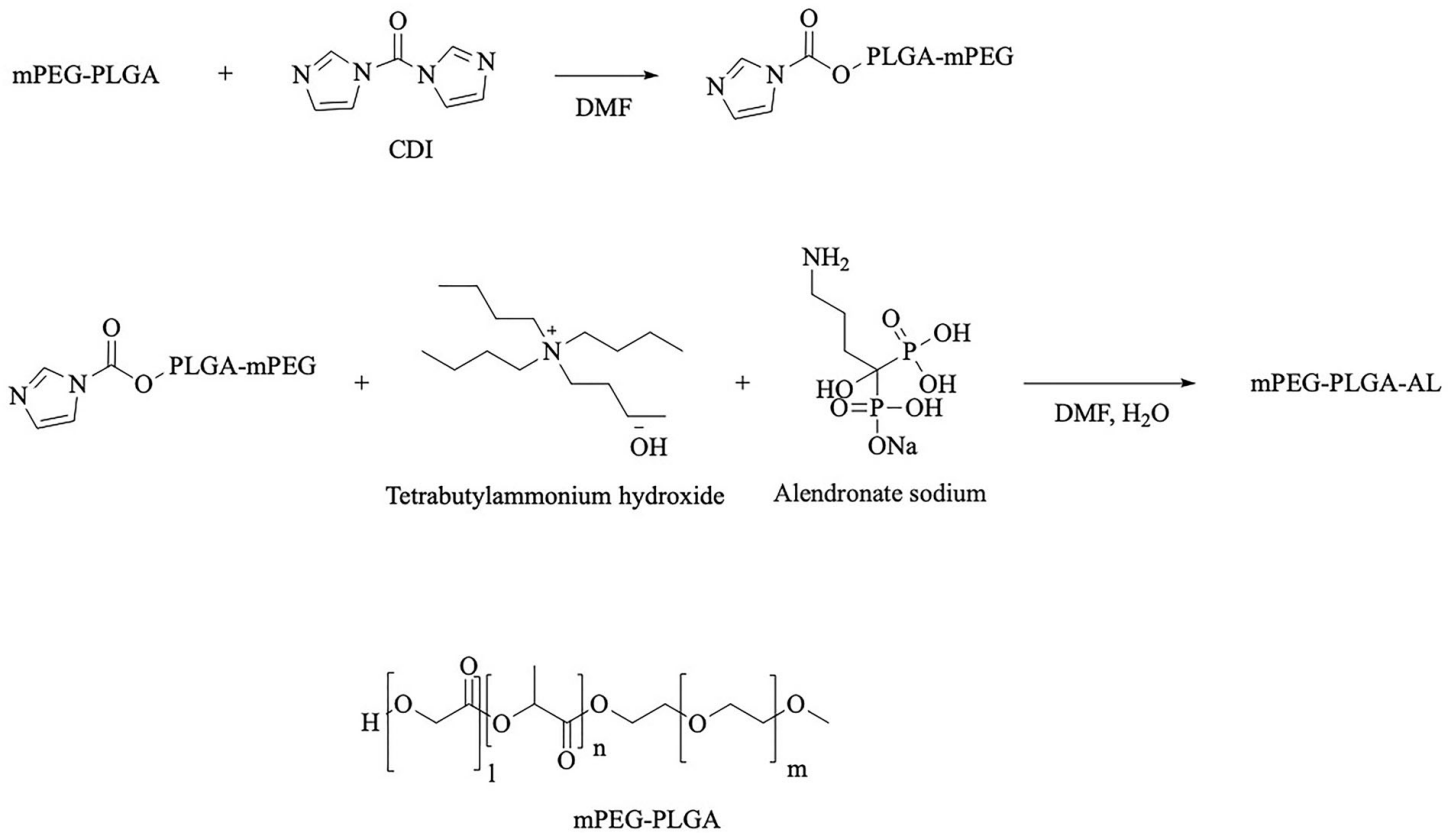
The structural formula of mPEG-PLGA and the synthetic route of AL-mPEG-PLGA.

Simply, the structural formula of mPEG-PLGA and the synthetic route of AL-mPEG-PLGA is shown in Figure 1. MPEG2000-PLGA18000 (50/50) compound (200 mg, 0.01 mmol) was dissolved in 2 mL DMF solution, and CDI (2.5 mg, 0.015 mmol) was added while stirring. The reaction was conducted for 5 h at room temperature. The reaction situation was monitored using a running board with a mixed solution of methylene chloride and methanol (methylene chloride: methanol = 15:1). After both compounds have reacted, a mixture of alendronate sodium trihydrate and tetrabutyl ammonium hydroxide solution (molar ratio =1:2) was added to the reaction solution under stirring, before it was allowed to react at 80 °C for 5 h. Next, the reaction was monitored using a running board with a mixture of dichloromethane and methanol (dichloromethane: methanol =15:1). Afterwards, DMF (70–80 °C) was removed with a rotary evaporator after the reaction. The remaining reaction solution was washed with a small amount of water, while the supernatant was removed by centrifugation (remove the excess sodium AL and tetrabutylammonium hydroxide). After that, the bottom white emulsion was dissolved with an appropriate amount of dichloromethane, but the product was separated and purified with a neutral Al_2_O_3_ column (dichloromethane: methanol = 30–15:1). The product quantity was 40 mg with a yield of 19.7%. Finally, hydrogen and phosphorus spectra (solvent deuterium chloroform) were applied to analyze the target compound.

### 2.4. HPLC analysis of AS

The concentration of AS was determined by an HPLC analysis method. An Agilent 1260 Infinity HPLC System (Agilent, USA) including an automatic injector and an ELSD detector was used. Chromatographic separation was carried out by Agilent Eclipse XDB-C18ODS column (4.6 mm × 250 mm, 5 μm, Waters, Ireland) at 30 °C. The mobile phase is comprised of acetonitrile and water (85:15, v/v). The drift tube temperature was 60 °C with the carrier gas volume flow rate of 1.7 mL/min. A series of AS standard solutions with different concentrations were prepared and analyzed with HPLC (*in vitro*, 10, 2, 1, 0.5, 0.2, 0.1 μg/mL; *in vivo*, 10, 5, 2, 1, 0.5, 0.2, 0.1 μg/mL). *In vivo* blank blood samples were treated by methanol precipitation of protein. The standard curves *in vitro* and *in vivo* for the determination of AS concentration were established respectively as follows:

$$Y=157.39X\;+\;104.75\;(R^{2}=0.9913)\;(In\;\mathrm{vitro})$$

$$Y=132.14X\;+\;82.497\;(R^{2}=0.9918)\;(In\;\mathrm{vivo})$$

where the *Y* means the peak area and the C denotes the AS concentration. The linear range of the standard curves *in vitro* and *in vivo* is 0.1–10 μg/mL.

### 2.5. Preparation of AS-AL-mPEG-PLGA

In this study, AS-AL-mPEG-PLGA was prepared by the dialysis method as described in the previous study (Song et al., 2020). Next, 10 mg of AL-mPEG-PLGA was precisely weighed and dissolved in 10 mL of distilled water. After 30 ultrasonic probes, 1.0 mg/mL of AL-mPEG-PLGA blank micelle solution was obtained. Afterwards, 15% of AS ethanol solution was gradually added to the solution under magnetic stirring. After 10 min of stirring, the solution was transferred to a dialysis bag (MWCO 7, 000, 000 Da), while ethanol was removed through dialysis with distilled water for 24 h. The dialyzed products were centrifuged at 4, 000 rpm/min for 10 min to remove the unencapsulated AS. After the supernatant was taken for freeze drying, AS-AL-mPEG-PLGA solid powder was prepared for sequential studies.

### 2.6. Characterization of AS-AL-mPEG-PLGA

#### 2.6.1. Particle size, zeta potential and morphology analysis

The physical characterizations of AS-AL-mPEG-PLGA, including the particle size and zeta potential, were measured with the NanoBrook 90 plus PALS instrument (Brookhaven Corporation, USA) which is based on principles of dynamic light scattering (DLS) and electrophoretic mobility at 25 °C. The whole measurements were measured 3 times in parallel.

Negative staining was applied to observe the morphology of AS-AL-mPEG-PLGA. Diluted micelles were dropped (micelles/H_2_O = 1:10) on a copper grid, prior to negatively staining for 30 s with acidic phosphotungstic (2%) solution. Next, the thin films were dried at room temperature for 30 min prior to observation via transmission electron microscope (TEM, JEM-2100, JEOL, Tokyo, Japan).

#### 2.6.2. Entrapment efficiency (EE) measurement

The EE of the preparation was determined based on the previous method (Wang et al., 2018). The prepared AS-AL-mPEG-PLGA aqueous dispersion solution (1 mL) was placed in an ultrafiltration centrifuge tube (30 KDa, Amicon Ultra-15, Millipore) and centrifuged at 10,000 rpm for 20 min at 4 °C to separate micelle-wrapped AS. After proper dilution of the filtrate and unfiltered samples with chromatographic methanol and filtration through a 0.22 µm microporous membrane filter, sample analysis was conducted according to the HPLC conditions stated under the section “HPLC analysis of AS.” Next, the peak area was recorded and then substituted into the standard curve to calculate the concentration of AS. The drug concentration in the filtrate after centrifugation indicates the encapsulated drug concentration. The drug concentration in non-centrifuged micelles represents the total drug concentration in micelles. Later, EE % was calculated by the following formula:

$$\mathrm{EE}\%=C_{1}/C_{2}\times 100\%$$

where *C*_1_ means the concentration of AS encapsulated in the micelle, and *C*_2_ denotes the total concentration of AS in the formulation.

### 2.7. Stability of AS-AL-mPEG-PLGA

AS-AL-mPEG-PLGA were stored at 4 and 25 °C for 30 days and accordingly sampled at 0, 15 and 30 days. Afterwards, the collected samples were centrifuged at 10,000 rpm for 10 min for the determination of drug content, EE and particle size.

### 2.8. Drug release study in vitro

The release of AS *in vitro* was determined by dialysis (Algul et al., 2018). AS free drug dispersion solution (5 mg/mL, 1 mL) and AS-AL-mPEG-PLGA containing the same volume and concentration of AS were added into dialysis bags (MV 3500 D, 25 mm × 5 m) accordingly. The dialysis bags were placed in a conical flask after their two sections have been fastened together. Subsequently, 100 mL releasing media (pH 1.2 HCl solution, double distilled water and pH 7.4 PBS solution) were added consequently, before the conical flask was placed in a thermostatic water bath oscillator (37 ± 0.5 °C, 100 rpm) for *in vitro* release experiment. Samples were taken from the conical flask after 0.083, 0.25, 0.5, 0.75, 1, 1.5, 2, 3, 4, 6, 8, 10, 12, 24, 36, 48, 60, 72, 84 and 96 h of oscillation, respectively, while the same stable release medium with the same volume was added accordingly to maintain its dynamic balance. The samples were centrifugated at 10,000 rpm for 10 min before injection into HPLC for analysis according to the chromatographic conditions under the section of “HPLC analysis of AS.” Finally, the peak areas were recorded, and the concentrations of AS were substituted into the standard curve to calculate the cumulative drug release rate.

### 2.9. In vitro cytotoxicity study

The cytotoxicity of the AS-AL-mPEG-PLGA was evaluated in MC3T3-E1 cells using the MTT method. In brief, MC3T3-E1 cells at the logarithmic growth stage were inoculated on 96-well plates at a density of 5 × 104 per well, then incubated at 37 °C and 5% CO_2_ for 24 h. After the cells were completely adherent to the wall, different concentrations (10, 25, 50, 100 and 200 μg/mL) of AL-mPEG-PLGA blank, AS-AL-mPEG-PLGA and mPEG-PLGA blank micellar solutions were added. At the same time, untreated blank cells were used as control, while 3 parallel groups were set for each well. After 48 h of continuous culture, we added 20 μL MTT solution (5 mg/mL) to each well. Later on, the culture was continued in the incubator for 4 h. After the supernatant was discarded, DMSO (100 μL) was added to each well at constant temperature oscillation for 20 min. The absorbance was measured (at 570 nm) with a microplate reader, wherein the cell survival rate was calculated through the following formula:

I%=(Atreat−Ablank)/(Acontrol−Ablank)×100%

where I% means cell viability rate, Atreat is the absorbance of the experimental group, Acontrol is the absorbance of the control group (without treatment) and the Ablank is the absorbance of medium (without any sample and seeded cell).

### 2.10. In vitro bone targeting evaluation

Hydroxyapatite (Hap) affinity of three micelles *in vitro* was investigated by labeling AL-mPEG-PLGA blank micelles, AS-AL-mPEG-PLGA and mPEG-PLGA blank micelles with octadecylamine-fluorescein isothiocyanate (ODA-FITC) fluorescence. The ODA-FTTC fluorescently labeled micelles were diluted into 1 mg/mL with deionized water, accordingly, prior to the determination of the initial fluorescence values of the micellar solution. Then, 4 mL of each micellar solution was added to 25 mg Hap. Next, the mixture was stirred slowly for 3 h by magnetic agitation until equilibrium under the condition of room temperature and dark light. After 3 h of equilibrium, the samples were centrifuged at 3000 r/min for 15 min to remove the sediment. The supernatant was determined using fluorescence spectrophotometer. The adsorption percentage of each micelle to Hap was calculated by the ratio of the fluorescence value of the superfluid to the initial fluorescence value after 3 h of Hap equilibrium.

### 2.11. Bone targeting efficacy in vivo

In order to further verify the bone targeting ability of AS-AL-mPEG-PLGA, the rats were given DIR fluorescent dye loaded AS-AL-mPEG-PLGA and mPEG-PLGA blank micelles via gavage. At 24 h after administration, the rats were sacrificed, and their bone was collected. In order to prepare AS-AL-mPEG-PLGA and mPEG-PLGA micelles loaded with fluorescent dye DIR, it was necessary to add DIR in methanol with an amount of 0.2% of polymer mass. Before pre-freezing, the micellar solution was centrifuged at 10,000 r/min for 5 min to remove free DIR. The other steps are the same as before. Later, the figures were photographed with *in vivo* imaging technique (DXS4000PRO, Kodak, USA).

### 2.12. Pharmacokinetic study in rats

Twelve SD rats (male, 200 ± 20 g) were maintained in the laboratory environment for 3 days before experiments. The rats were randomly divided into the free AS group and AS-AL-mPEG-PLGA group with 6 rats in each group. The rats have fasted from food for 12 h with free access to water before the experiment. Rats in the two groups were orally given free AS (dispersed CMC-Na solution) and AS-AL-mPEG-PLGA of equivalent AS dose (150 mg/kg). At each point of 0.25, 0.5, 0.75, 1, 1.5, 2, 2.5, 3, 4, 6, 8, 10, 12 and 24 h after administration, blood samples (0.4 mL) were collected from the orbit of rats with capillaries. The blood samples were placed in a 1.5 mL EP tube filled with heparin sodium and centrifuged at 3700 rpm for 10 min. Aliquot (200 μL) of supernatant was collected into another clean 1.5 mL EP tube. Next, 600 μL of methanol was added into the tube before vortexing for 1 min. The mixture was centrifuged at 10,000 rpm for 10 min and the methanol layer was completely removed prior to drying with N_2_ at 37 °C. The residue was dissolved with 100 μL methanol coupled with vortexing for 1 min, and then 10 min of centrifugation at 10,000 rpm. Then, the supernatant was taken and analyzed with HPLC. The peak areas of AS and internal standard were recorded and substituted into the standard curve *in vivo* to subsequently calculate the concentration of AS.

### 2.13. Detection of as content in rats’ bones

After the pharmacokinetic experiment, the rats were sacrificed and the femurs were homogenized with normal saline at a ratio of 1 g/mL. We then centrifugated the homogenized samples at 10,000 rpm for 10 min, before treatment of the supernatant (200 μL) via the same method as described in the section “Pharmacokinetic study in rats.” Finally, the samples were analyzed via the HPLC method to detect the AS concentration in rats’ bones.

### 2.14. Anti-osteoporotic effect in rats

#### 2.14.1. Ovariectoporosis model establishment

Bilateral ovariectomy (OVX) was performed to establish the rat osteoporosis model according to earlier work (Shakir et al., 2015). Next, 24 rats were randomly divided into four groups (*n* = 6): sham operation group (Sham), OVX model group (OVX), free AS group (AS) and AS-AL-mPEG-PLGA group. Rats were anesthetized by intraperitoneal injection of pentobarbital sodium (1%, 8 mL/kg). Later, the rats were placed on a sterile table and fixed prone, while the surgical site on the back was shaved and sterilized with iodophor. Double incisions were taken under the costal back to separate the muscular layer and retroperitoneum into the abdominal cavity. The ovaries were found in the adipose tissue below the kidneys and were removed with bent tweezers and ligated before complete excision of the bilateral ovaries. After confirming that there was no active bleeding, the muscle layer and skin were interlocked, whilst the wound was disinfected. In the Sham group, only a small amount of adipose tissue around the ovaries was removed without damage to both ovaries. The operation was done gently while attention was paid to the sterility principle. After the operation, the rats were kept warm, while drug administration began 4 weeks afterwards at once a day for 12 weeks. Rats in the groups Sham and OVX were given 2 mL of normal saline via the oral route. Rats in the AS and AS-AL-mPEG-PLGA groups orally received 2 mL of free AS (dispersed in CMC-Na solution, 150 mg/kg) and AS-AL-mPEG-PLGA solution (150 mg/kg) every day, respectively.

#### 2.14.2. Serum biochemical analysis

All the rats were sacrificed and blood samples were collected. The serum levels of TRACP-5b, BGP, IL-6 and CTX-1 were determined by enzyme-linked immunosorbent assay (ELISA) to evaluate bone absorption and bone formation. The operation steps were strictly in accordance with the instructions of the kits. Finally, the absorbances of the samples were measured at 450 nm with the microplate reader, so as to calculate the concentration of each biochemical index in the serum.

#### 2.14.3. Micro-computerized tomography (CT) analysis

In order to evaluate the bone microstructure, the right femur was analyzed with high-resolution micro-CT using the micro-CT imaging system (Siemens, Inveon) according to the method described in the literature (Zhang et al., 2012). The femur was fixed in the centrifuge tube by the long axis, while the surrounding was filled with gauze. Quantitative parameters of bone microstructure including percent bone volume (BV/TV), trabecular thickness (Tb.Th), trabecular number (Tb.N), trabecular separation (Tb.Sp), structural model index (SMI) and bone mineral density (BMD) were measured. All the digital data, 2 D and 3 D images were provided by the built-in software of micro-CT (Siemens, Inveon).

#### 2.14.4. Histological analysis

The femur was fixed with 4% paraformaldehyde, decalcified with EDTA (10%, pH7.4) and embedded in paraffin. Longitudinal serial sections (4 μm) were mounted on microscope slides coated with polylysine. Hematoxylin and eosin (H&E) staining was conducted according to the manufacturer’s procedure. The staining area was photographed by ImageJ software.

### 2.15. Statistical analysis

The measurement data were expressed as mean ± standard deviation with statistical analysis performed via SPSS13.0 software. Student’s *t* test was used to assess the statistical significance and *P* < .05 was considered a significant difference.

## 3. Results and discussion

### 3.1. Synthesis of AL-mPEG-PLGA

**Figure 2.**
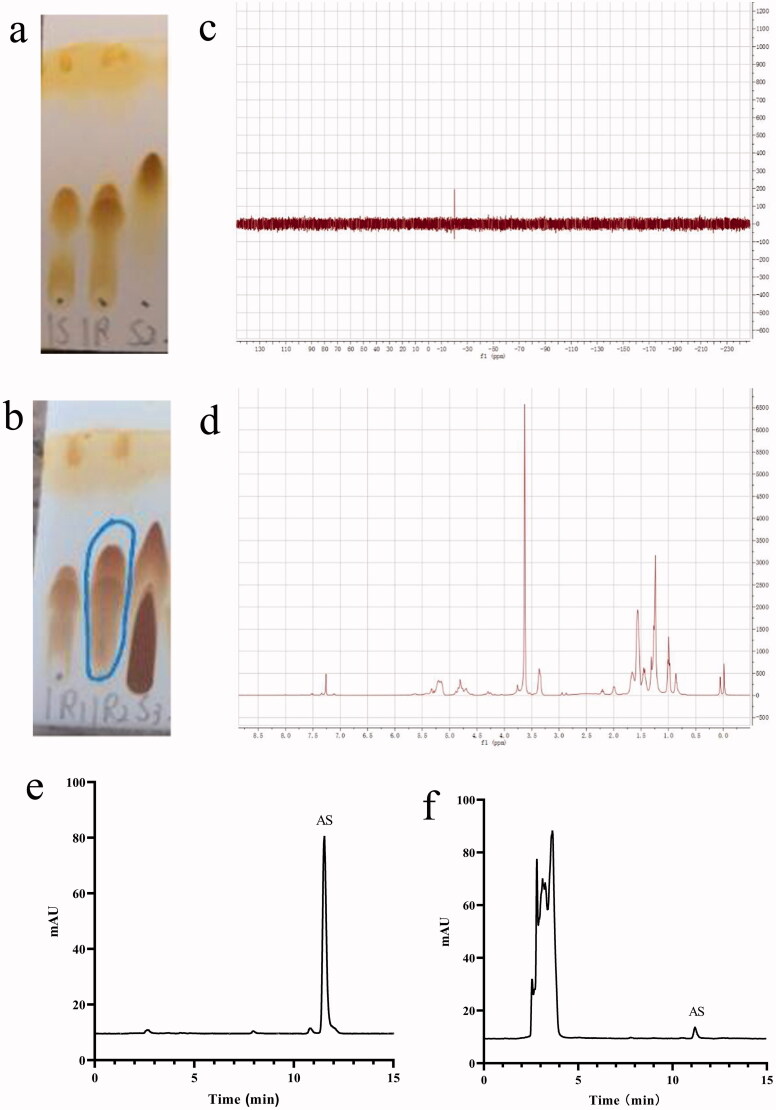
(a) Running board monitoring of the first step reaction. 1S was the control of mPEG_2000_-PLGA_18000_ (50/50) and 1 R was the reaction solution of the first step reaction, S2 was the CDI. (b) Running board monitoring of the second step reaction. 1R1 was the reaction solution of the first step, 1R2 was the reaction solution of the second step and S3 was the solution of tetrabutylammonium hydroxide. (c) Phosphorus spectrum of the target product. (d) Hydrogen spectrum of the target product. (e) HPLC chromatogram of AS *in vitro*. (f) HPLC chromatogram of AS *in vivo*.

As shown in Figure 2(a), there was no obvious CDI raw material in the reaction solution, thus indicating that the first step reaction was basically complete. As displayed in Figure 2(b), the reaction process cannot be directly judged according to the running situation of the silicone plate. Since the polarity of impurities in the raw material tetrabutylammonium hydroxide after running was similar to that of the suspected product (indicated by the blue box), the unreacted tetrabutylammonium hydroxide was removed by washing with water in the post-treatment. The polarity of the other raw material alendronate sodium was greater, while only a certain number of impurities in water solubility were observed, which we tried to remove by washing with water.

The structure of the product was analyzed by phosphorus and hydrogen spectra, wherein the results are shown in Figure 2(c,d). The characteristic peaks of each monomer can be found in the target product, which suggests the target product was bonded successfully. The results are consistent with existing literature (Pignatello et al., 2009).

### 3.2. HPLC study of AS

The HPLC diagram of AS *in vitro* and *in vivo* is presented in Figure 2(e,f). Under the chromatographic conditions and systems, the retention time of AS was about 11.5 min with a good peak shape and well-separated solvent peak. Moreover, endogenous substances in blank plasma exhibited no interference in the analysis and detection of AS. Therefore, this chromatographic method could be used for *in vivo* and *in vitro* content determination of AS.

### 3.3. Characterization of AS-AL-mPEG-PLGA

**Figure 3.**
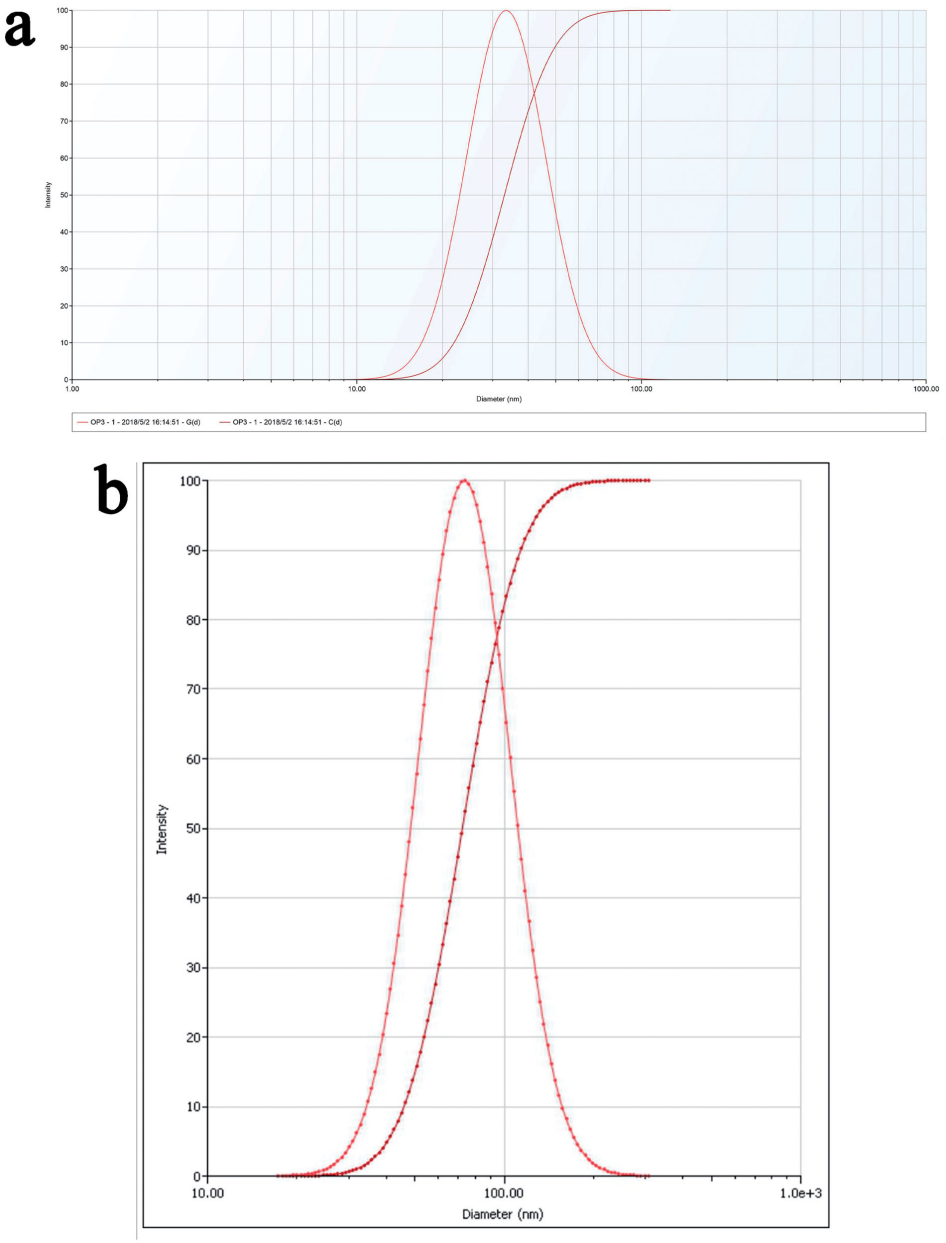
(a) Particle size distribution diagrams of AL-mPEG-PLGA blank micelle. (b) Particle size distribution diagrams of AS-AL-mPEG-PLGA.

**Figure 4.**
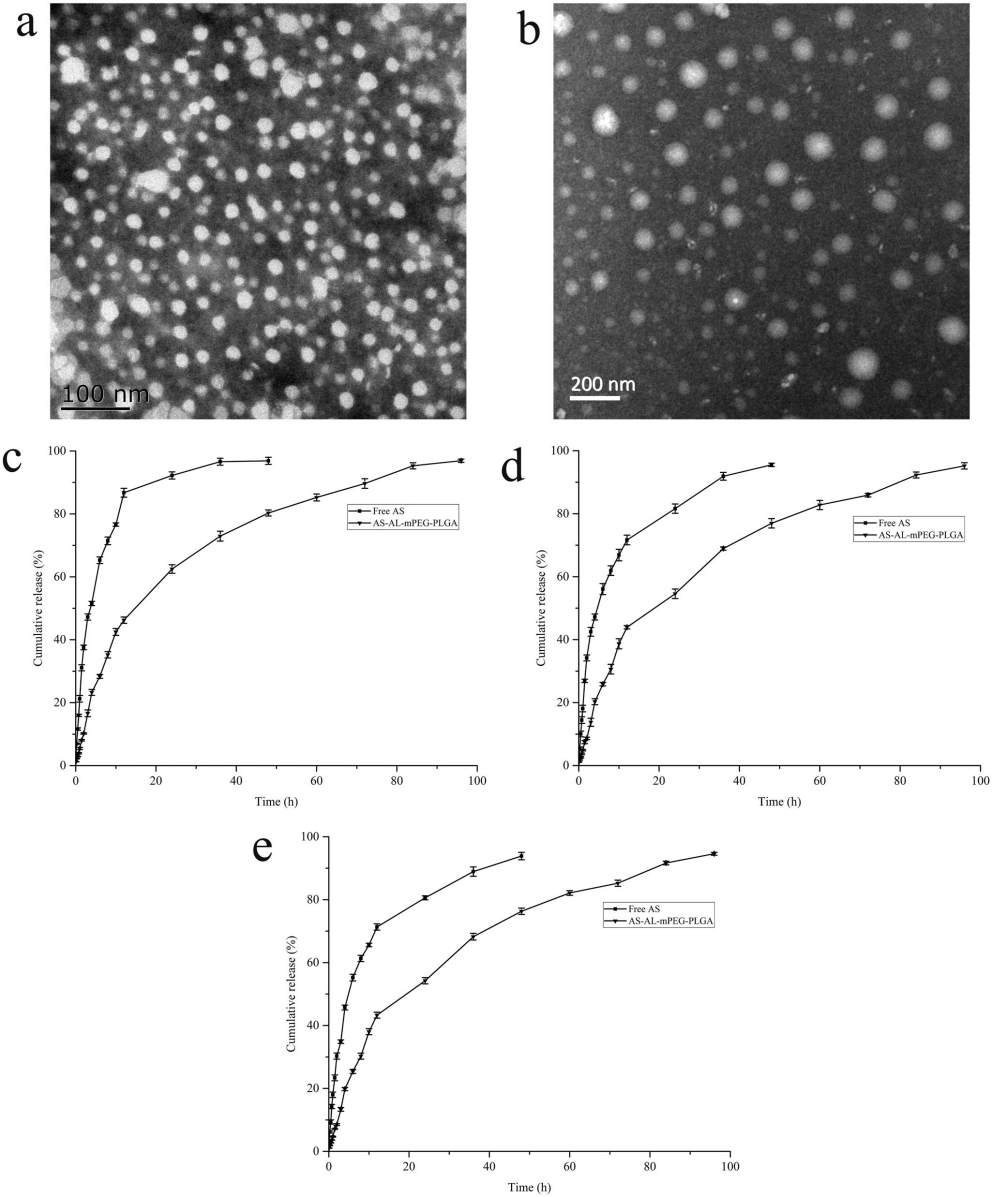
(a) Transmission electron microscope (TEM) image of AL-mPEG-PLGA blank micelles. (b) TEM image of AS-AL-mPEG-PLGA. (c) *In vitro* release curves of free AS and AS-AL-mPEG-PLGA in pH 1.2 HCl solution. (d) *In vitro* release curves of free AS and AS-AL-mPEG-PLGA in double distilled water. (e) *In vitro* release curves of free AS and AS-AL-mPEG-PLGA in pH 7.4 PBS solution.

The average particle size and zeta potential of the prepared AS-AL-mPEG-PLGA were 73.3 ± 3.8 nm and −23.02 ± 0.51 mV, respectively. Also, the average particle size and zeta potential of the AL-mPEG-PLGA blank micelle were 35.2 ± 1.4 nm and −23.46 ± 0.31 mV, respectively. Accordingly, the particle size distribution diagrams of AS-AL-mPEG-PLGA and AL-mPEG-PLGA blank micelle are shown in Figures 3(a,b). Under TEM, AS-AL-mPEG-PLGA and AL-mPEG-PLGA blank micelles were uniform in size and spherical in shape (Figure 4(a,b)) amid the particle size being consistent with the DLS results. Previous research has reported that the particle size of nanocarriers is an important factor that usually affects the circulation of the encapsulated drug (Cai et al., 2010). In general, formulations with relatively high absolute zeta potential are considered to be relatively stable to prevent the possibility of coalescence and thus maintain the uniformity of nanodroplets (Zhao et al., 2012). Therefore, smaller particle sizes and higher absolute zeta potential in this study were beneficial to the drug delivery of AS.

Besides, EE% of AS-AL-mPEG-PLGA was 96.16 ± 0.18%. This finding indicated that higher amounts of AS were entrapped in the AS-AL-mPEG-PLGA. Thus, the high EE% of nanocarriers is a significant index for the improvement of the oral bioavailability of drugs (Ong et al., 2016). The synthesized AL-mPEG-PLGA could form self-assembled micelles and core-shell structures in the aqueous medium. The reason is that PEG, as a hydrophilic shell, has good micellar stability in the self-assembly process, while PLGA could improve AS solubility (Ling et al., 2013). Thus, this strategy of loading AS into tissue targeting carriers can enhance the therapeutic potential of the drug.

### 3.4. Stability of AS-AL-mPEG-PLGA

After 30 days at 4 and 25 °C, the appearance of AS-AL-mPEG-PLGA did not change significantly. It could be seen from Table 1 that the drug content, EE and diameter of AS-AL-mPEG-PLGA did not significantly alter after 30 days of storage at 4 and 25 °C. It could be preliminarily concluded that the AS-AL-mPEG-PLGA exhibited good stability at 4 and 25 °C, which was consistent with the results of zeta potential.

**Table 1.** Stability of AS-AL-mPEG-PLGA at different temperatures (mean ± SD, *n* = 3).

Day	Drug content (%)		EE (%)		Diameter (nm)	
	4 °C	25 °C	4 °C	25 °C	4 °C	25 °C
0	12.38 ± 0.67	12.33 ± 0.81	96.23 ± 0.91	96.31 ± 0.73	73.66 ± 1.89	74.18 ± 1.11
15	12.31 ± 0.71	12.41 ± 0.72	96.12 ± 0.81	95.69 ± 0.68	74.23 ± 1.89	75.27 ± 1.53
30	12.17 ± 0.74	12.16 ± 0.88	95.63 ± 0.88	94.55 ± 0.69	75.29 ± 2.15	76.81 ± 1.79

AS-AL-mPEG-PLGA, alendronate modified mPEG-PLGA loaded with astragaloside nano-micelles; EE, encapsulation efficiency.

### 3.5. *In vitro* as release study

The *in vitro* release curves of AS from AS-AL-mPEG-PLGA in pH 1.2 HCl solution, double distilled water and pH 7.4 PBS solution were evaluated for 96 h. As shown in Figure 4(c–e), the cumulative release rates of free AS in pH1.2 HCl solution, double distilled water and pH 7.4 PBS solution were 96.87%, 93.85% and 95.95% respectively within 48 h, while the release time of AS from AS-AL-mPEG-PLGA could be extended to 96 h, with the respective cumulative release rates in three media being 96.91%, 94.60% and 95.19%. These results suggested that AS-AL-mPEG-PLGA showed a good sustained release effect. In addition, no significant difference was observed in the cumulative release rate between free AS and micelles, which implies that AL-mPEG-PLGA did not affect the drug release rate. This may be due to two stages of drug release in AS-AL-mPEG-PLGA group. The first stage was the initial rapid release (0–48 h), while the second stage was the extended time up to 48–96 h (Wang et al., 2019). In the first 48 h, more than 76% of AS was released from the polymeric micelles in all the three dissolution media, amid the drug release rate reaching more than 94% in the final time interval (48–96 h). The slow release in the later stage might be due to the hydrophobic drug (AS) being encapsulated in the core of the micelles, which requires a diffusion process, amid the drug molecules being gradually released from the core of polymeric micelles, thus delaying the drug release time. The two-stage release mode could transport AS into bone and ensure that enough drug concentration reaches the osteoporotic site. As a result, loading AS into AS-AL-mPEG-PLGA could reduce the drug intake of patients, thereby reducing the possible adverse reactions of patients in the future. Xie et al. (2018) studied the release of simvastatin from tetracycline-mediated mPEG-PLGA micelles. The results showed that simvastatin micelles released rapidly at the initial stage, after which the sustained release lasted for more than 72 h. Yao et al. (2014) prepared minocycline-loaded mPEG-b-PLA micelles (MIN-mPEG-b-PLA) and studied their release in PBS. It was found that almost 100% of the minocycline was released from free minocycline within 24 h. However, the cumulative release of minocycline in MIN-mPEG-b-PLA was more than 14 days Therefore, the use of micellar drug delivery system may avoid burst drug release coupled with delayed release and reduced intake of the drug.

### 3.6. *In vitro* cytotoxicity assay

**Figure 5.**
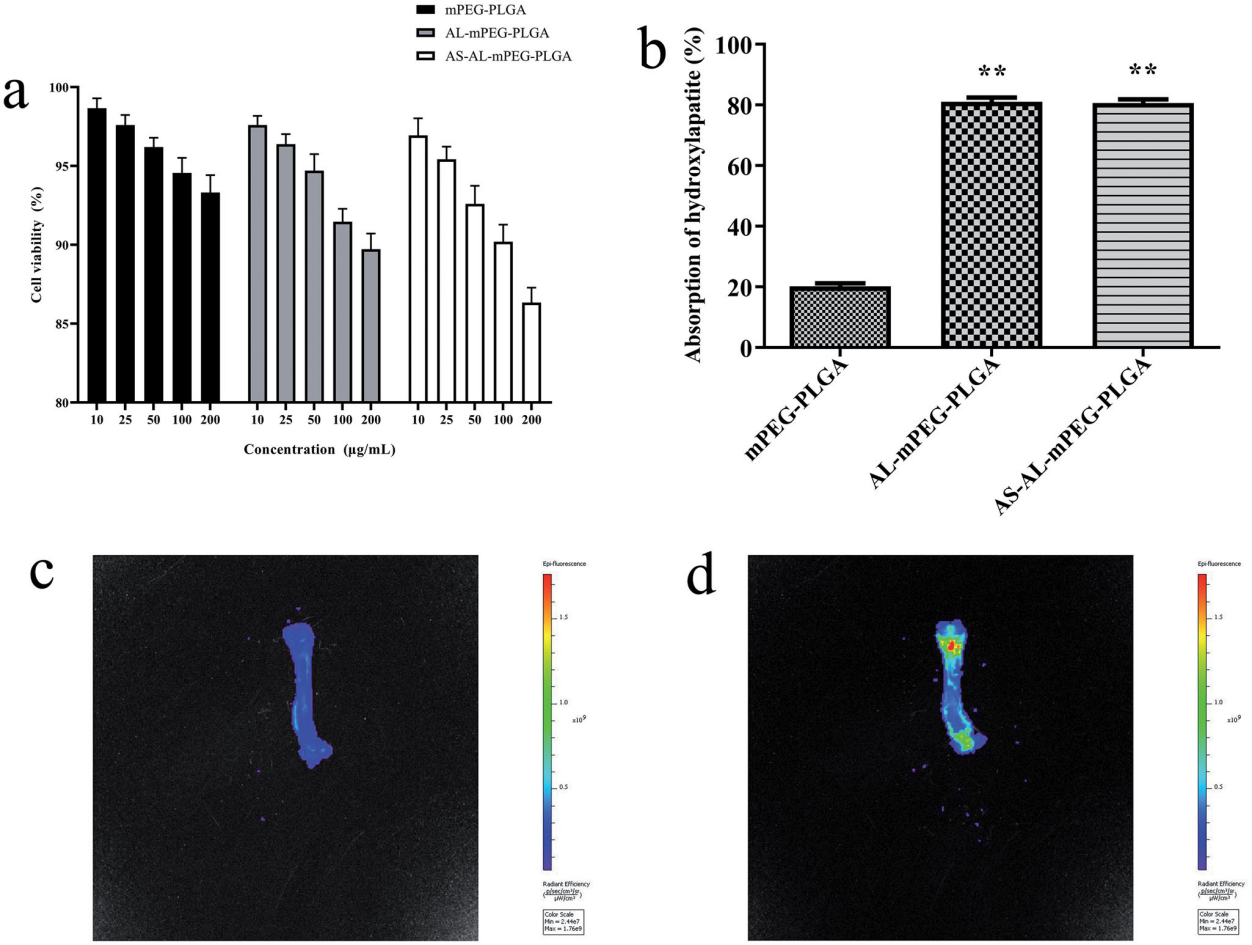
(a) Cell viability of MC3T3-E1 cells *in vitro*. (b) Hydroxyapatite adsorption affinity experiments results. (c) *In vivo* imaging images of rats were given with mPEG-PLGA micelles. (d) *In vivo* imaging images of rats were given with AS-AL-mPEG-PLGA. *P < .05, compared with mPEG-PLGA group, **P < .01, compared with mPEG-PLGA group.

AS-AL-mPEG-PLGA, as a drug delivery carrier, should have good biocompatibility. In this regard, the MTT assay was used to evaluate the cell safety of AS-AL-mPEG-PLGA (Du et al., 2010). Within a certain concentration range (10–200 μg/mL), compared with mPEG-PLGA, AL-mPEG-PLGA blank micelles exhibited no significant effect on cell viability, thereby indicating that AL modified mPEG-PLGA had no toxic effect on MC3T3-E1 cells (Figure 5(a)). AS-AL-mPEG-PLGA displayed a negligible effect on cells in the concentration range of 10–200 μg/mL. But even at the concentration of 200 μg/mL, the cell viability of MC3T3-E1 cells that were affected by AS-AL-mPEG-PLGA was still up to 85%, which suggests that AS-AL-mPEG-PLGA had good biocompatibility within a certain concentration range.

### 3.7. *In vitro* bone targeting evaluation

High binding activity to bone tissue is a prerequisite for bone-targeted drug delivery. Hap is the main component of the bone matrix, which is an ideal specific binding site for the bone-targeted nano-drug delivery system. Compounds tend to be deposited in bone and incorporated into hydroxyapatite and have the binding ability with bone calcium (Xie et al., 2018). Therefore, any molecule with a specific targeting ability to hydroxyapatite can be used as a guide or carrier of bone-targeted drugs, so that drugs can selectively act on bone tissue. Notably, AL has a high affinity for Hap. After entering the human body, AL was found to be mainly deposited in the active sites of bone metabolism, wherein it demonstrated an active targeting effect (Polymeri et al., 2015). Therefore, in this study, mPEG-PLGA, which has the ability to avoid RES phagocytosis (Petros & DeSimone, 2010), was modified with AL which could act as bone-targeted therapy (Pignatello et al., 2009). As shown in Figure 5(b), a significant difference in adsorption quantity was found between the mPEG-PLGA and AS-AL-mPEG-PLGA groups. The proportion of AL-mPEG-PLGA blank micelles and Hap-bound AS-AL-mPEG-PLGA reached 81%, while the percentage of mPEG-PLGA blank micelles as the control group on Hap was only 21.18%, which indicated that AL modified micelles drastically increased the adsorption capacity of Hap (*P* < .01). This finding may be ascribed to good bone targeting and affinity of AL.

### 3.8. *In vivo* bone distribution of AS-AL-mPEG-PLGA

The distribution of the micelles in the bone of SD rats was observed to explore its bone targeting ability *in vivo*. As displayed in Figure 5(c,d), the femurs of rats that received DIR-loaded AS-AL-mPEG-PLGA showed strong fluorescence compared with those that received DIR-loaded mPEG-PLGA micelles. This result was consistent with the *in vitro* Hap experiment. AL modified micelles could bind Hap with higher affinity than mPEG-PLGA micelles. Therefore, AS-AL-mPEG-PLGA exhibited good bone tissue targeting ability, which was beneficial to the targeted transport of AS to bone tissues.

### 3.9. Pharmacokinetic study

**Figure 6.**
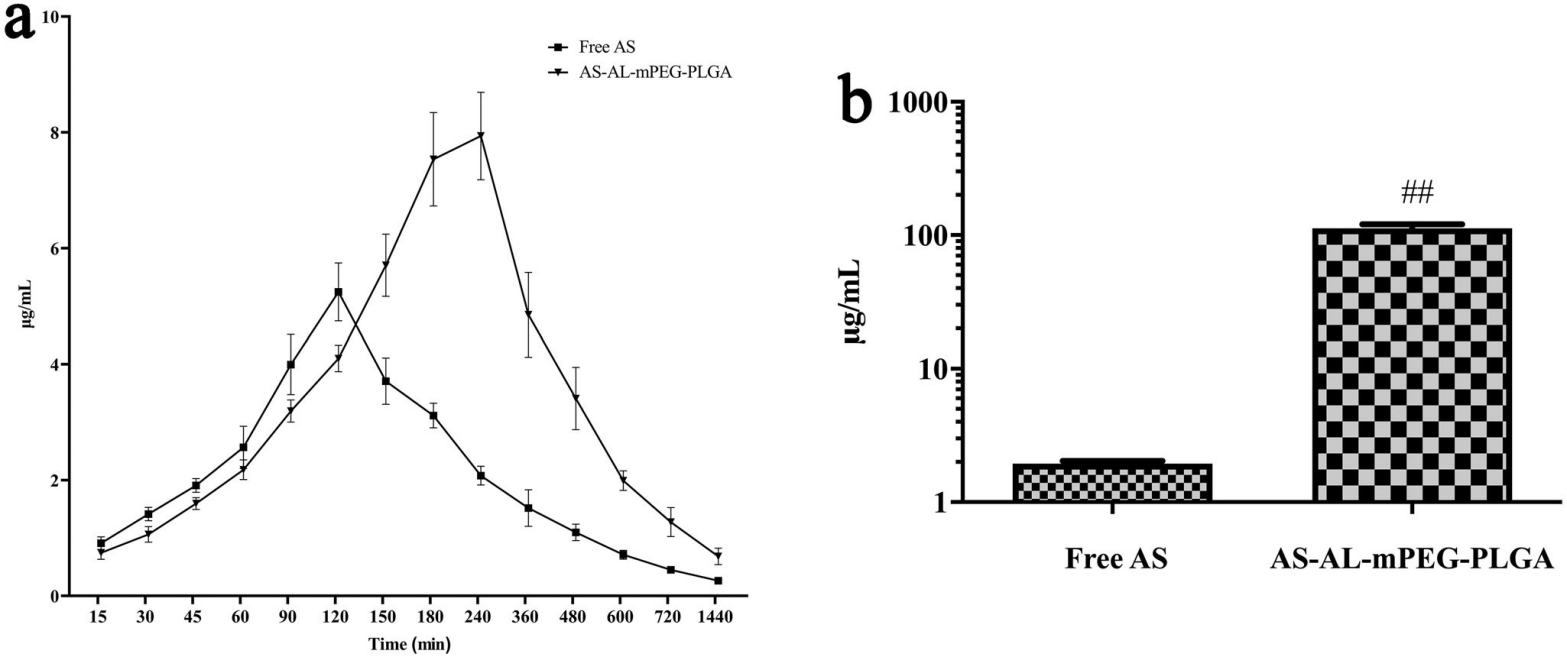
(a) Plasma concentration-time profiles of AS after oral administration of free AS and AS-AL-mPEG-PLGA. (b) Determination of AS content in rats’ bones. ##*P* < .01, compared with the free AS group.

According to the measured blood concentration data, the corresponding blood concentration-time curves were drawn, wherein the result is shown in Figure 6(a). Table 2 summarizes the relevant pharmacokinetic parameters including time to reach maximum plasma concentration (*T*_max_), maximum plasma concentration (*C*_max_), half-life (*t*_1/2_), area under the concentration-time curve (AUC) and mean residence time (MRT). It is obvious that the plasma concentration of free AS increased continuously after oral administration to reach the *C*_max_ of 5.25 ± 0.50 μg/mL at 120 min. However, the plasma concentration decreased rapidly after 120 min with the mean plasma concentration of the free AS group being only 0.26 ± 0.05 μg/mL at 1440 min. The data showed that AS exhibited poor absorption coupled with fast metabolism after oral administration. In contrast, after oral administration of AS-AL-mPEG-PLGA, *C*_max_ in the rats was significantly increased to 7.94 ± 0.76 μg/mL (*P* < .01), while *T*_max_ was prolonged to 240 min (*P* < .01). Compared with free AS, the AUC0-t of AS-AL-mPEG-PLGA increased from 1520.45 ± 45.19 to 3556.32 ± 282.46 min·μg/mL (*P* < .01). When AS was entrapped in AS-AL-mPEG-PLGA, the MRT and *T*_1/2_ increased from 333.00 ± 8.57 min and 395.71 ± 26.44 min to 409.47 ± 14.18 min and 957.75 ± 188.90 min (*P* < .01), respectively, suggesting that AS-AL-mPEG-PLGA could reduce the rapid clearance of AS and prolong the circulation time of AS, as well as promote the accumulation of increased AS amount in bone tissue. Most importantly, the relative bioavailability of AS-AL-mPEG-PLGA was 233.90% compared with free AS. These results indicated that AS-AL-mPEG-PLGA maintained good integrity and stability in the systemic circulation, prevented the metabolism and elimination of AS to the greatest extent, amid a significant increase in the oral bioavailability of as the drug. It is possible that after mPEG-PLGA modification, the steric hindrance of AS-AL-mPEG-PLGA became larger. Meanwhile, the conditioning effect with plasma protein became smaller, while the probability of being captured by the reticuloendothelial system became lower (Kraft et al., 2014), thus prolonging the circulation time of AS *in vivo*, thereby contributing to better efficacy of AS. The results agreed with previous studies regarding the oral administration of mPEG-PLGA formulations (Rawat et al., 2016; Hasanpour et al., 2021).

**Table 2.** Major pharmacokinetic parameters of free AS and AS-AL-mPEG-PLGA (mean ± SD, *n* = 6).

Parameters	Free AS	AS-AL-mPEG-PLGA
*C*_max_ (μg/mL)	5.25 ± 0.50	7.94 ± 0.76^##^
*T*_max_ (min)	120 ± 0	240 ± 0^##^
AUC_0-t_ (min·μg/mL)	1520.45 ± 45.19	3556.32 ± 282.46^##^
*T*_1/2_ (min)	395.71 ± 26.44	957.75 ± 188.90^##^
MRT (min)	333.00 ± 8.57	409.47 ± 14.18^##^

##*P* < .01, compared with free AS group. AS, astragaloside; AS-AL-mPEG-PLGA, alendronate modified mPEG-PLGA loaded with astragaloside nano-micelles.

### 3.10. Detection of as content in rats’ bones

AS concentration was detected in bone after oral administration of free AS and AS-AL-mPEG-PLGA in rats. The concentrations of AS were determined through the aforementioned HPLC method, and the results are depicted in Figure 6(b). The concentration of AS in the free AS group was 1.95 ± 0.17 μg/mL, which was significantly lower than that in AS-AL-mPEG-PLGA group (112.78 ± 15.36 μg/mL) (*P* < .01). The results showed that increased accumulation of AS in bone tissue after oral administration of AS-AL-mPEG-PLGA may be due to better bone targeting tenacity of the nano-micelle, which was beneficial to improving the anti-osteoporotic effect of AS. Thus, the nano-carrier benefited from the higher affinity of AL for bone tissues (Polymeri et al., 2015).

### 3.11. Anti-osteoporotic effect of AS-AL-mPEG-PLGA

**Figure 7.**
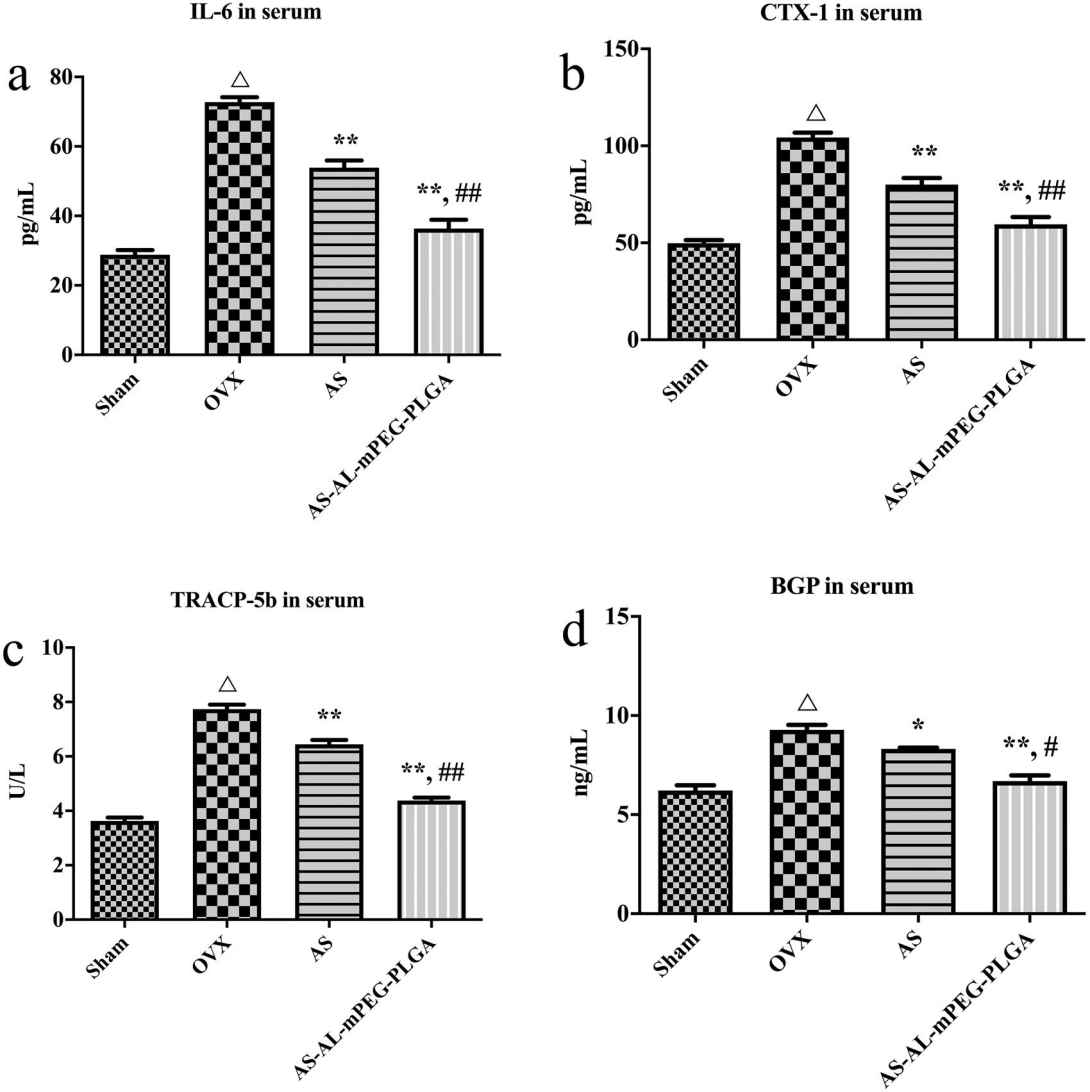
Serum levels of IL-6 (a), CTX-1 (b), TRACP-5b (c) and BGP (d) in osteoporotic rats of Sham, OVX, AS, and AS-AL-mPEG-PLGA groups. ^△^*P* < .01, compared with Sham group, *P < .05, compared with OVX group, **P < .01, compared with OVX group, ^##^*P* < .05, compared with AS group, ^##^*P* < .01, compared with AS group.

To investigate the beneficial effect of AS-AL-mPEG-PLGA on osteoporosis, the serum levels of IL-6, CTX-1, TRACP-5b and BGP were detected through the ELISA method. One of the most important pathways in the regulation of bone metabolism is the involvement of cytokines. In particular, IL-6 is one of the bone resorptions stimulating factors. It acts on the precursor cells of osteoclasts, induces them to differentiate into osteoclasts, and plays a role in bone resorption during reconstruction (Kimble et al., 1996). When the bone marrow microenvironment was stimulated by estrogen deficiency, IL-6 cooperated with other bone resorption factors and receptors to promote the resorption of bone (Suda et al., 1995). Figure 7(a) showed that in comparison with group Sham, the expression of IL-6 in the OVX group was markedly increased (*P* < .01), which indicated that after OVX, estrogen level was decreased, thereby inducing osteoblasts to secrete IL-6 which in turn increased the formation of osteoclasts, thus enhancing bone resorption and eventually leading to osteoporosis (Udagawa et al., 2000). After administration of free AS and AS-AL-mPEG-PLGA, the expression level of IL-6 decreased significantly (*P* < .01), which may be mediated by regulating the secretion of estrogen to produce the anti-osteoporotic effect (Pfeilschifter et al., 2002). Moreover, AS-AL-mPEG-PLGA remarkably increased it from 53.88 ± 4.23 pg/mL to 36.40 ± 4.97 pg/mL in comparison with AS, respectively (*P* < .01). Importantly, the process of bone metabolism could be reflected by changes in biochemical indicators of bone metabolism. As shown in Figure 7(b–d), the serum levels of CTX-1, TRACP-5b and BGP showed a remarkable difference between Sham vs. OVX (*P* < .01), wherein BGP was secreted by osteoblasts. Suggestively, the main function of BGP is to maintain the normal mineralization rate of bone and inhibit the formation of abnormal apatite crystals (Chopin et al., 2012). Notably, BGP level in serum could directly reflect the osteocyte activity and bone formation in patients with osteoporosis (Niimi et al., 2014). Moreover, TRACP-5b is an important biochemical indicator of bone resorption. The level of TRACP-5b in serum may reflect the activity of bone cells and the state of bone resorption (Wang et al., 2019). The CTX-1 is an important material for the formation of bone fibers and a significant marker of bone formation (Lehrskov et al., 2020). Therefore, the rats’ model of osteoporosis was successfully established. The levels of CTX-1, TRACP-5b and BGP in serum were significantly decreased by AS and AS-AL-mPEG-PLGA compared with OVX group (*P* < .01). Moreover, there were significant differences in serum CTX-1, TRACP-5b and BGP levels between AS and AS-AL-mPEG-PLGA groups (*P* < .01, *P* < .01 and *P* < .05, respectively). These data suggested that AS-AL-mPEG-PLGA could enhance the effect of AS in reducing serum levels of bone metabolism-related indicators.

**Figure 8.**
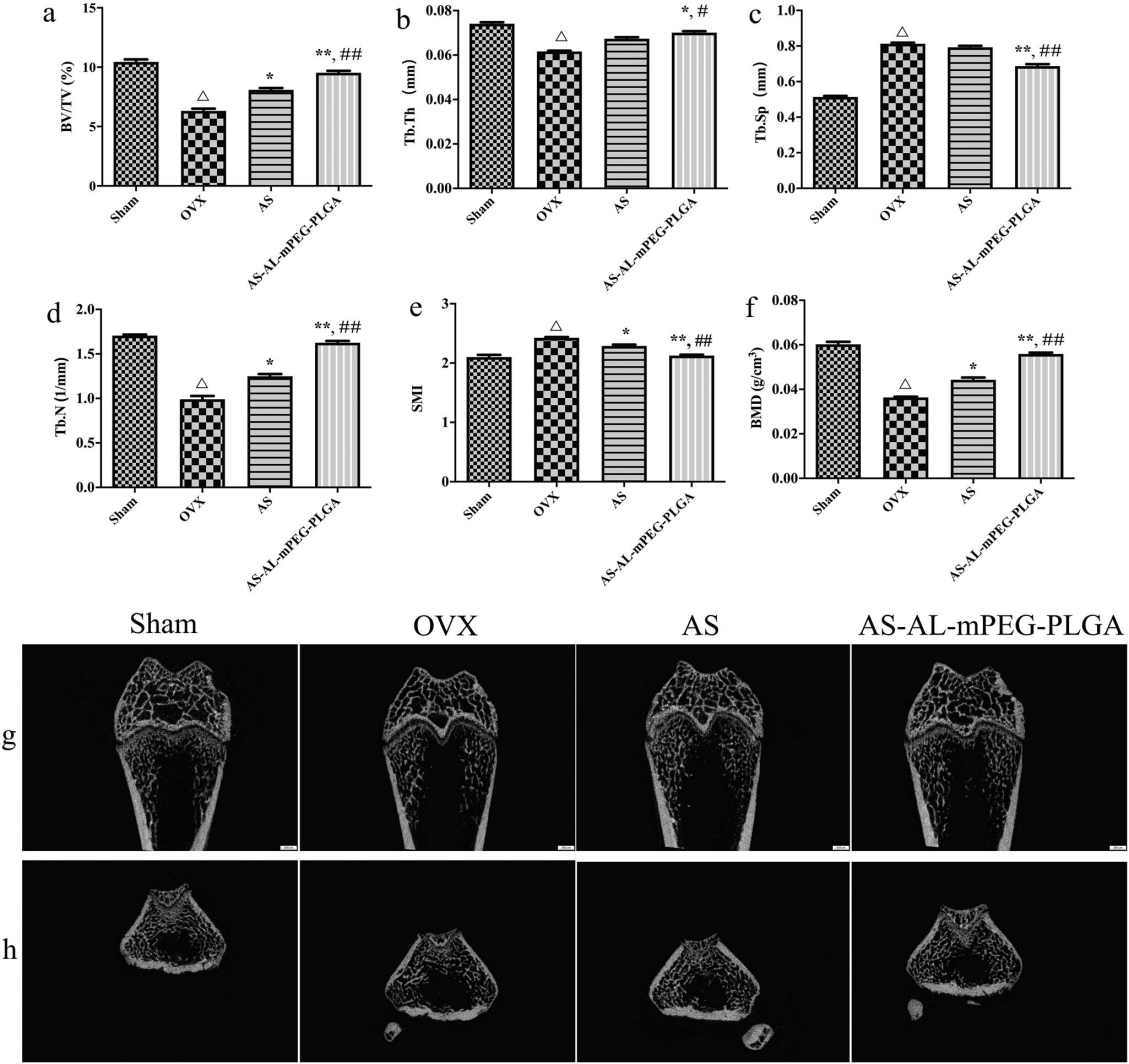
Micro-CT results of osteoporosis rats in Sham, OVX, AS, and AS-AL-mPEG-PLGA groups. (a–f) The quantitative characterization index including BV/TV, Tb.Th, Tb.N, Tb.Sp, SMI and BMD. (g) 2-dimensional micro-CT images and (h) 3-dimensional micro-CT images of the femurs. ^△^*P* < .01, compared with Sham group, *P < .05, compared with OVX group, **P < .01, compared with OVX group, ^##^*P* < .05, compared with AS group, ^##^*P* < .01, compared with AS group.

In the quantitative analysis of micro-CT scanning results, there were significant differences in BV/TV, Tb.Th, Tb.N, Tb.Sp, SMI and BMD between OVX and Sham groups (*P* < .01), which demonstrate a successful induction of the osteoporotic model in SD rats. The bone microstructure is one of the parameters to evaluate bone strength (Liu et al., 2013). The decrease in bone mechanical strength is an essential feature of osteoporosis, whereas structural changes in trabecular bone play an important role in bone strength. Therefore, a comprehensive study of trabecular microstructure has become a key focus of bone health research. In addition, BMD is also a key parameter to drive bone strength. In clinical practice, BMD is widely used as a predictor of fracture risk (Park & Na, 2013). SMI reflected the characteristics of trabecular plate-shaped structures or rod-shaped structures. When osteoporosis occurs, the trabecular bone changes from a plate-shaped structure to a rod-shaped structure, while the value of SMI increases (Effendy et al., 2017). As shown in Figure 8(a–f), after the OVX rats were treated with AS or AS-AL-mPEG-PLGA, the BV/TV, Tb.Th, Tb.N and BMD were notably increased, but the Tb.Sp and SMI substantially reduced. In particular, statistical comparisons indicated that AS group exhibited more excellent restoring ability compared with the AS-AL-mPEG-PLGA group. Thus, we suggested that AS may play its therapeutic role by restoring bone microstructure and repairing damaged bone tissue. The outcomes of a decrease in bone metabolism markers by nano-carriers were supported by enhanced bone parameter. These results indicated that AS-AL-mPEG-PLGA aided AS to restore the bone microarchitecture of rats with osteoporotic symptoms.

The 3 D and 2 D reconstruction images were measured with micro-CT assay, wherein the results are the same as the above-mentioned finding (Figure 8(g,h)). In comparison with the Sham group, the OVX cohort demonstrated significant trabecular bone loss and decreased BMD, thus leading to osteoporosis. Compared with the OVX group, both AS and AS-AL-mPEG-PLGA could significantly reduce osteoporosis and increase dense bone mass, which suggests a substantial improvement in bone microstructure and volume. As expected, AS-AL-mPEG-PLGA further enhanced the health of trabecular bone and obtained the best microstructure in micro-CT analysis, which was close to the Sham group.

**Figure 9.**
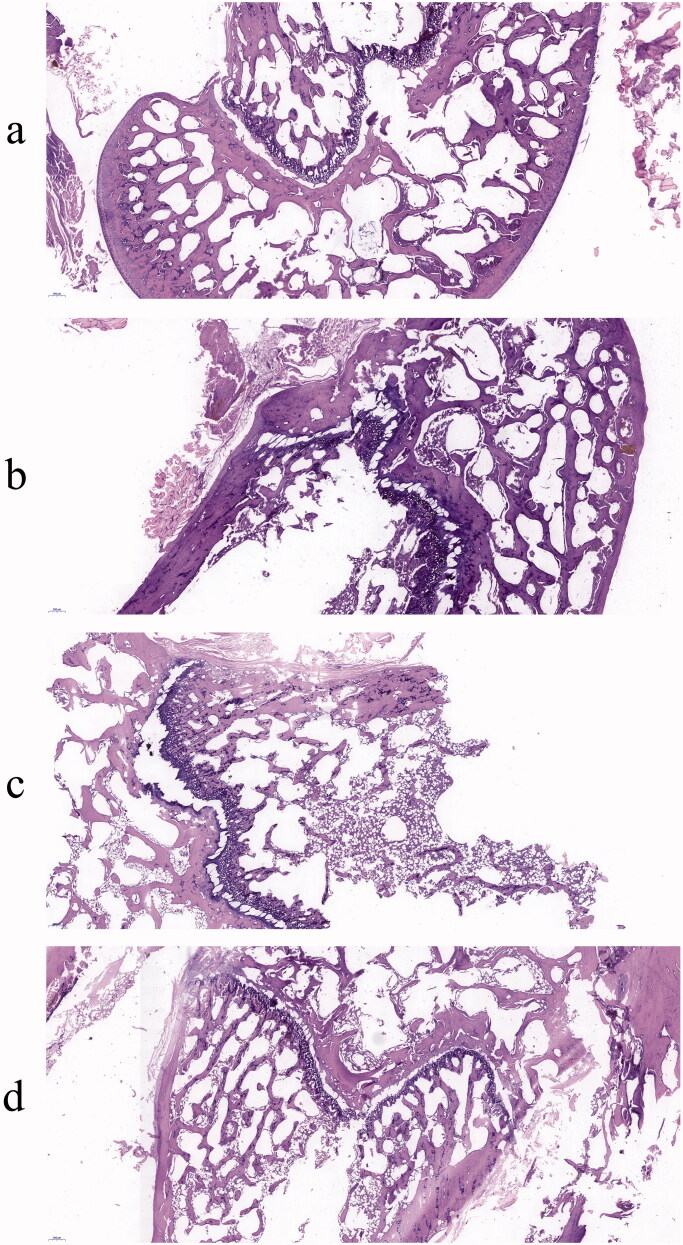
Histological analysis of femur from rats of Sham (a), OVX (b), AS (c), and AS-AL-mPEG-PLGA (d) groups.

H&E staining results are shown in Figure 9. In the Sham group, the density of femur and trabecular bone was normal. Compared with the Sham group, the number of adipose tissues in the OVX group was higher with the trabecular bone getting thinner while the trabecular space became wider. AS and AS-AL-mPEG-PLGA groups displayed relatively less adipose tissue compared with the OVX group. The trabecular bone and BMD were much more prominent in AS-AL-mPEG-PLGA group which showed more resemblance to the Sham group, amid consistency with the micro-CT results. Therefore, AS-AL-mPEG-PLGA increased the ability of AS to resist trabecular bone loss and restored bone microstructure in OVX rats. In all the experiments, the performance of AS-AL-mPEG-PLGA was better than that of free AS, which provided decisive evidence for the good bone targeting ability of AS-AL-mPEG-PLGA and its positive role in managing osteoporosis. The underlying reason for this finding may be attributed to the sustained-release effect of AS-AL-mPEG-PLGA and improved oral bioavailability, wherein, the accumulation and targeting effect of AL in bone may play a significant role in targeting the potential of AS-AL-mPEG-PLGA (Pan et al., 2008), thus increasing the efficacy of its anti-osteoporotic activity (Li et al., 2015).

## 4. Conclusion

The aim of this study was to construct AL-mPEG-PLGA loaded AS micelles and evaluate their physicochemical properties and anti-osteoporotic activity. AL-mPEG-PLGA was successfully synthesized with mPEG-PLGA and AL. Through the determination of particle size, morphology and EE, it was found that the particle size of AS-AL-mPEG-PLGA was about 45 nm. The size of micelles was uniform with a spherical shape and concomitant high EE. *In vitro* release studies confirmed that AS-AL-mPEG-PLGA demonstrated a significant sustained-release effect. In addition, related experiments showed that AS-AL-mPEG-PLGA exhibited acceptable biocompatibility in the range of 10–200 μg/mL coupled with good bone tissue targeting ability. Moreover, the micelle could significantly increase the oral bioavailability of AS and delay the internal circulation time. Furthermore, AS-AL-mPEG-PLGA showed significantly better therapeutic efficacy than free AS in osteoporosis rats. Therefore, the AS drug delivery system could be used as a novel and effective candidate for the treatment of osteoporosis.

## Data Availability

The data presented in this study are available upon request from the corresponding author.
